# Supplementary material for: Smart Cancer-Targeting and Super-Sensitive Sensing of Eu3+/Tb3+-Induced Hyaluronan Characteristic Nano-Micelles with Effective Drug Loading and Release
Source: Molecules. 2024 Oct 26;29(21):5070. doi: 10.3390/molecules29215070 (PMC11547788; doi:10.3390/molecules29215070)
Supplement: Supplementary file 1 [file molecules-29-05070-s001.zip › molecules-3245785-supplementary.pdf]

Supporting information

Smart Cancer-Targeting and Super-Sensitive Sensing of Eu^{3+}/Tb^{3+}-Induced Hyaluronan Characteristic Nanomicelles with Effective Drug Loading and Releasing

Yupeng Bi[1], Longlong Li[1], Jin Liu[1], Yao Wang[1], Boying Wang[1], Christopher D. Snow[2], Jun Li[3], Matt J. Kipper[*,2], Laurence A. Belfiore[1,2], Jianguo Tang*[,1]

[1]Institute of Hybrid Materials, National Center of International Joint Research for Hybrid Materials Technology, National Base of International Sci. & Technology Cooperation on Hybrid Materials, Qingdao University, 308 Ningxia Road, Qingdao 266071, People's Republic of China.

[2]Department of Chemical and Biological Engineering, School of Materials Science and Engineering, and School of Biomedical Engineering, Colorado State University, Fort Collins, Colorado 80523, USA

[3]School of Materials Science and Engineering, Shanghai University of Engineering Science, Shanghai 201620, China

*Corresponding author, e-mail: tang@qdu.edu.cn, Matthew.Kipper@ColoState.EDU

Contents:

Figure S1: (a) relative contents of elements in sample A tested by EDS, (b) relative contents of elements in sample G tested by EDS.

Figure S2: (a) UV absorption curves of DTIC with different concentrations. (b) UV absorption curves of a set of DTIC in vitro release solution. (c) UV absorption-concentration curves of DTIC.

Figure S3: In the malignant melanoma animal model, a continuous intratumorally injection of PBS buffer (1), ENMs (2), TNMs (3), ENMs loaded with DTIC (4), TNMs loaded with DTIC (5), and free drugs (6) was administered for 10 days. Photographs of tumors from six sample groups

Table S1: Relative tumor volume & Tumor Growth Inhibition value

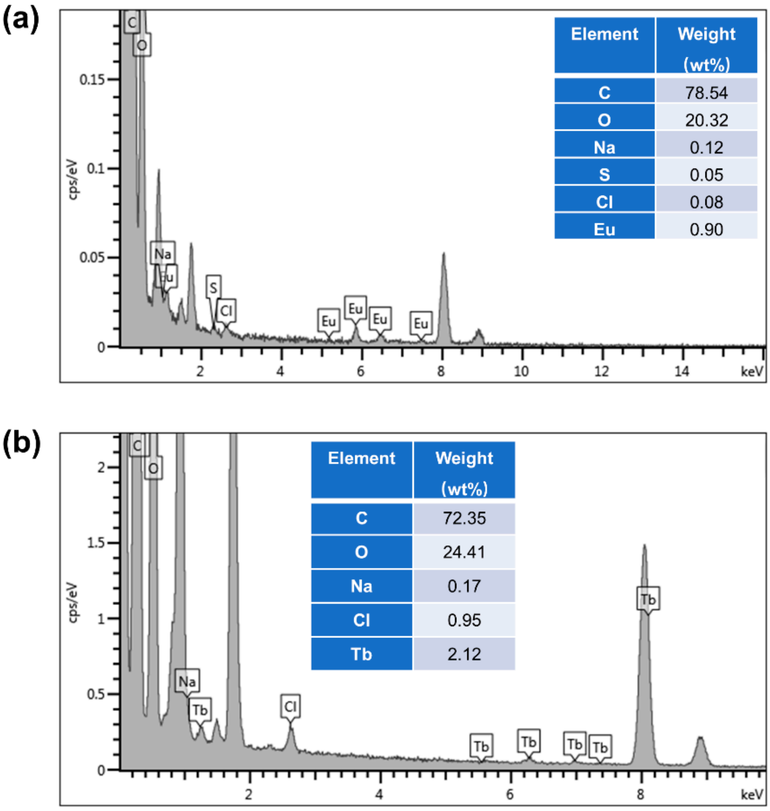

Figure S1

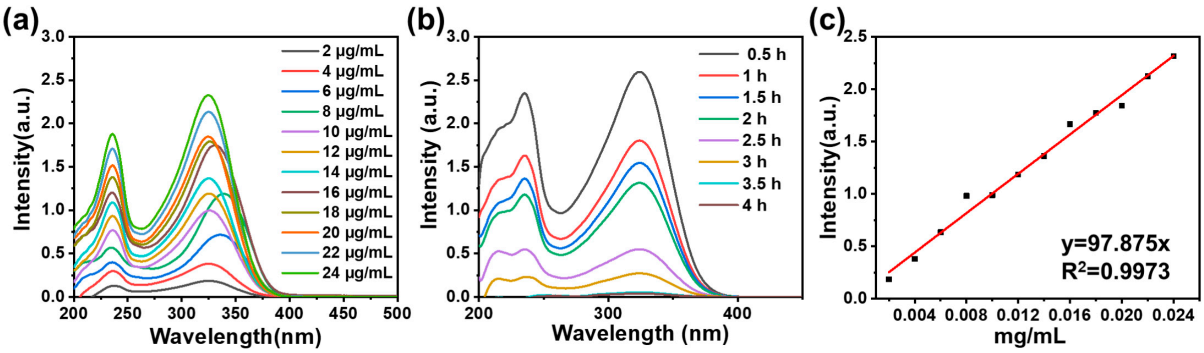

Figure S2

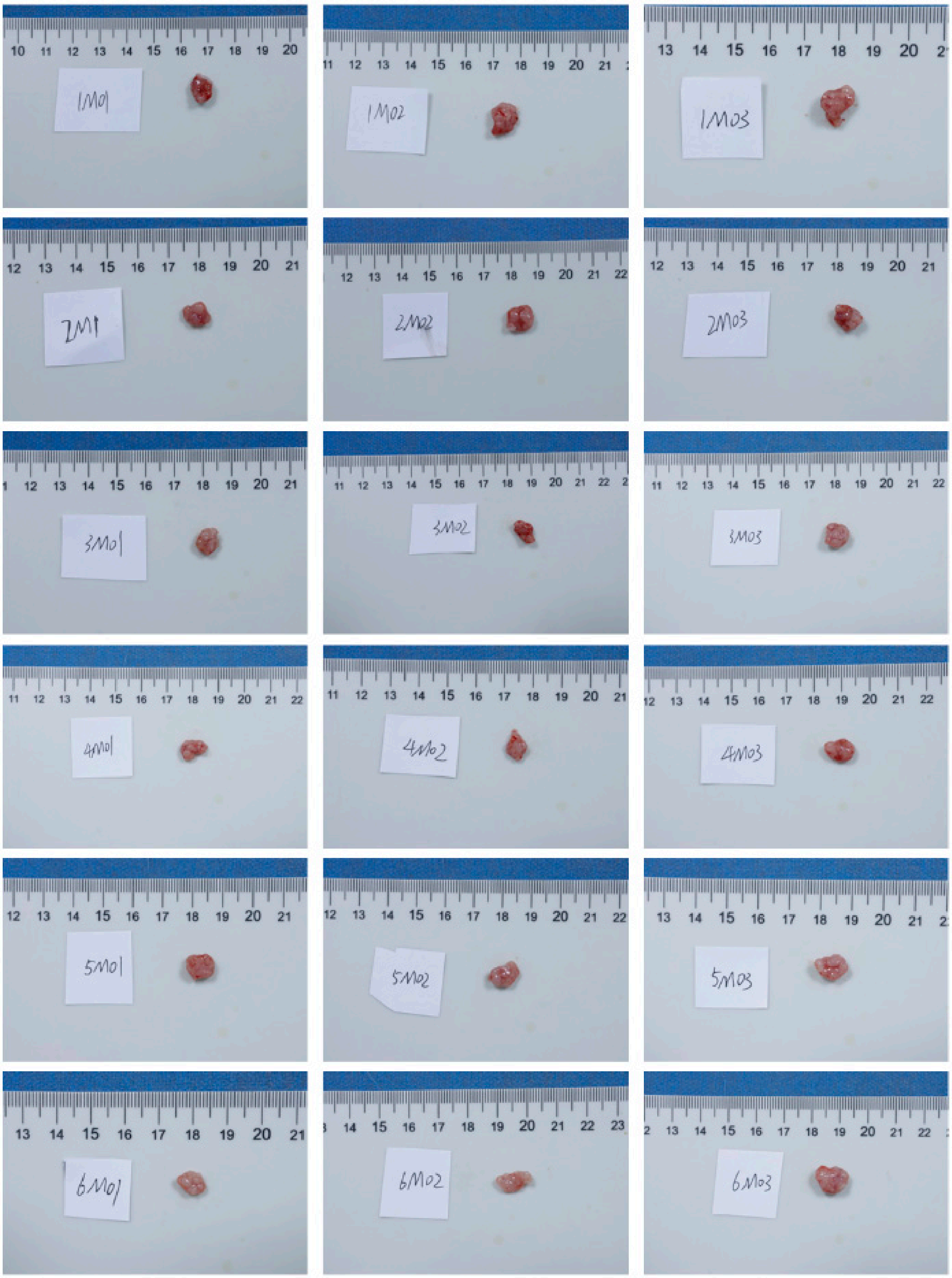
1M01
1M02
1M03
2M1
2M02
2M03
3M01
3M02
3M03
4M01
4M02
4M03
5M01
5M02
5M03
6M01
6M02
6M03

Figure S3

Relative tumor volume & Tumor Growth Inhibition value

Control

Group \ Incubation days	0	1	2	3	4	5	6	7	8	9	10
1M01	1	1.121	1.706	1.833	1.897	2.037	2.083	2.364	2.813	3.449	3.789
1M02	1	1.158	1.547	1.659	1.784	2.126	2.198	3.007	4.16	5.09	5.876
1M03	1	1.046	1.047	1.213	1.632	1.852	2.173	2.494	3.514	3.669	4.029
Mean	1	1.108	1.433	1.568	1.771	2.005	2.151	2.622	3.496	4.069	4.565
SD	0	0.047	0.281	0.261	0.109	0.114	0.049	0.278	0.55	0.727	0.932
TGI	/	/	/	/	/	/	/	/	/	/	/

ENMs

Group \ Incubation days	0	1	2	3	4	5	6	7	8	9	10
2M01	1	1.014	1.153	1.214	1.346	1.511	1.817	2.177	2.701	2.911	3.575
2M02	1	1.016	1.178	1.299	1.388	1.793	1.955	2.137	3.241	3.413	4.475
2M03	1	1.017	1.13	1.178	1.229	1.369	1.721	1.709	1.96	2.204	3.162
Mean	1	1.016	1.154	1.23	1.321	1.558	1.831	2.008	2.634	2.843	3.737
SD	0	0.001	0.02	0.051	0.067	0.176	0.096	0.212	0.525	0.496	0.548
TGI	0	0.084	0.195	0.215	0.254	0.223	0.149	0.234	0.247	0.301	0.181

TNMs

Group \ Incubation days	0	1	2	3	4	5	6	7	8	9	10
3M01	1	1.006	1.08	1.18	1.254	1.401	1.49	1.534	1.757	2.35	3.319
3M02	1	1.005	1.18	1.177	1.179	1.32	1.322	1.76	2.048	2.207	3.032
3M03	1	1.009	1.033	1.101	1.148	1.368	1.902	2.509	2.808	3.481	4.831
Mean	1	1.007	1.098	1.153	1.194	1.363	1.571	1.934	2.204	2.68	3.727
SD	0	0.002	0.061	0.037	0.045	0.033	0.244	0.417	0.443	0.57	0.789
TGI	0	0.092	0.234	0.265	0.326	0.32	0.27	0.262	0.369	0.341	0.183

ENMs + DTIC

Group \ Incubation days	0	1	2	3	4	5	6	7	8	9	10
4M01	1	1.106	1.106	1.228	1.176	1.7	1.596	1.669	2.261	2.435	3.941
4M02	1	1	1.046	1.019	0.974	1.012	1.174	1.517	1.923	2.26	3.171
4M03	1	1.053	1.122	1.223	1.267	1.482	1.615	1.815	2.422	2.759	3.263
Mean	1	1.053	1.091	1.156	1.139	1.398	1.462	1.667	2.202	2.485	3.458
SD	0	0.043	0.032	0.097	0.122	0.287	0.204	0.122	0.208	0.207	0.344
TGI	0	0.05	0.238	0.263	0.357	0.303	0.321	0.364	0.37	0.389	0.242

TNMs + DTIC

Group \ Incubation days	0	1	2	3	4	5	6	7	8	9	10
5M01	1	1.044	1.13	1.322	1.362	1.601	1.669	2.218	2.421	3.177	3.407
5M02	1	1.037	1.094	1.281	1.221	1.431	1.558	1.725	2.028	2.3	3.032
5M03	1	0.971	1.039	1.1	1.101	1.267	1.176	1.456	1.986	2.554	3.159
Mean	1	1.017	1.088	1.235	1.228	1.433	1.468	1.8	2.145	2.677	3.199
SD	0	0.033	0.037	0.096	0.107	0.136	0.211	0.316	0.196	0.369	0.156
TGI	0	0.082	0.241	0.213	0.306	0.285	0.318	0.314	0.386	0.342	0.299

DTIC

Group \ Incubation days	0	1	2	3	4	5	6	7	8	9	10
6M01	1	0.999	1.143	1.259	1.328	1.608	1.842	2.629	3.051	3.547	4.076
6M02	1	1.01	1.12	1.177	1.239	1.509	1.621	2.003	2.614	3.408	3.808
6M03	1	1.124	1.336	1.423	1.5	1.713	2.082	2.155	2.571	2.776	3.198
Mean	1	1.045	1.2	1.286	1.356	1.61	1.848	2.262	2.746	3.243	3.694
SD	0	0.057	0.097	0.102	0.108	0.083	0.188	0.266	0.217	0.335	0.367
TGI	0	0.058	0.163	0.18	0.235	0.197	0.141	0.137	0.215	0.203	0.191

Table S1
